# The vaccination acceptance, confidence, and conviction on influenza in the Middle East, Eurasia, and Africa among healthcare providers (VACCIMENA-HCP) project 2023: Determinants of vaccination behavior

**DOI:** 10.1016/j.ijregi.2025.100572

**Published:** 2025-01-19

**Authors:** Mine Durusu Tanriover, Gaelle Vallee-Tourangeau, Valentin A. Kokorin, Vera N. Larina, Mouna Maamar, Hicham Harmouche, Oğuz Abdullah Uyaroğlu, Dilan Yağmur Kutlay, Jalila Ben Khelil, Abdul-Azeez A. Anjorin, Muhammad Suleman Rana, Jabrayil Jabrayilov, Fatima Al Slail, Dalal Al Kathiry, Hasina Al Harthi, Ramy Mohamed Ghazy, Milad Gahwagi, Alireza Mafi, Parvaiz Koul, Salah Al Awaidy

**Affiliations:** 1 Hacettepe University Vaccine Institute, Ankara, Türkiye; 2 Kingston Business School, Kingston University, Kingston upon Thames, United Kingdom; 3 Peoples' Friendship University of Russia named after Patrice Lumumba, Department of Hospital Therapy with courses in Endocrinology, Hematology, and Clinical Laboratory Diagnostics, Moscow, Russian Federation; 4 Pirogov Russian National Research Medical University (RNRMU), Department of Polyclinic Therapy, Moscow, Russian Federation; 5 Mohamed V University Faculty of Medicine, Department of Internal Medicine, Geriatric Unit, Rabat, Morocco; 6 Mohamed V University Faculty of Medicine, Department of Internal Medicine, Rabat, Morocco; 7 Hacettepe University Faculty of Medicine, Ankara, Türkiye; 8 University of Tunis EI Manar Faculty of Medicine, Abderrahmen Mami Hospital, Medical Intensive Care Unit, Ariana, Tunisia; 9 Lagos State University, Department of Microbiology (Virology Research), Ojo, Nigeria; 10 National Institute of Health, Department of Virology, Islamabad, Pakistan; 11 Ege Hospital, Department of Nephrology, Baku, Azerbaijan; 12 Directorate of the National Diabetes Prevention and Control Program, Ministry of Health, Riyadh, Saudi Arabia; 13 Royal Hospital, Department of Research Section, Muscat, Oman; 14 High Institute of Public Health, Alexandria University, Department of Tropical Health, Alexandria, Egypt; 15 Benghazi University Faculty of Medicine, Department of Obstetrics and Gynecology, Benghazi, Libya; 16 Sanofi Vaccines, Istanbul, Türkiye; 17 Sher-i-Kashmir Institute of Medical Sciences, Srinagar, India; 18 Office of Health Affairs, Ministry of Health, Muscat, Oman

**Keywords:** Influenza, Vaccination, Healthcare workers, Hesitancy, ME'NA-ISN

## Abstract

**Objectives:**

Annual vaccination is the most effective way to prevent and control the health and economic burden of seasonal influenza. Healthcare providers (HCPs) play a crucial role in vaccine acceptance and patient advocacy. This study aimed to explore the barriers and drivers of HCPs’ vaccine acceptance and advocacy for the vaccination of their patients in countries in the Middle East, Eurasia, and Africa.

**Methods:**

Healthcare providers from 10 countries (Azerbaijan, Egypt, Libya, Morocco, Nigeria, Pakistan, Russia, Saudi Arabia, Tunisia, and Türkiye) were surveyed using opportunity sampling between 20^th^ December 2022, and 1^st^ March 2023. The previously developed motors of influenza vaccination acceptance (MoVac-Flu) and engagement with vaccination advocacy (MovAd) scales were used.

**Results:**

In the final sample of 721 responses, Russia was the leading country, enrolling 37% of all respondents. Sentiment cluster profiles were labeled as “engaged” or “hesitant” and “confident” or “diffident.” Overall, 28% demonstrated hesitancy and 30.5% demonstrated diffident sentiments toward self-vaccination behavior. HCPs exhibited very low rates of routine vaccination and infrequent advocacy of seasonal influenza vaccinations. In contrast, those who felt engaged with influenza vaccination were more likely to be vaccinated themselves and found it easier to incorporate influenza vaccination in their practice. Those who felt engaged in influenza vaccination also felt more confident in advocating for it than those who felt hesitant. The evidence suggests that the strength of the relationship between engagement and confidence varies across countries.

**Conclusions:**

This study provides insights into the behavioral patterns of HCPs regarding their own influenza vaccination and advocacy for patient vaccination. Cluster membership was a strong predictor of vaccination behaviors and attitudes, although regional differences were observed. For hesitant and diffident HCPs, tailored strategies to increase awareness and empower them regarding vaccines and vaccine-preventable diseases are crucial.

## Highlights

• Healthcare providers (HCPs) play a crucial role in vaccine acceptance and advocacy.
• Barriers and drivers of HCPs vaccine acceptance and advocacy were explored.
• Sentiment cluster profiles were “engaged”/“hesitant” and “confident”/“diffident.”
• Hesitant HCPs exhibited low rates of influenza vaccination and advocacy.
• Engaged HCPs more likely to vaccinate themselves and their patients.

## Introduction

Seasonal influenza affects approximately 1 billion people worldwide annually. Among these, 3-5 million cases result in severe illness. This disease is responsible for 290,000-650,000 respiratory deaths annually [1]. Annual vaccination is essential for combating seasonal influenza and reducing its impact on public health and the economy.

Healthcare providers (HCPs) have a pivotal role in increasing vaccine acceptance and advocating for their patients. By receiving the vaccine themselves, HCPs not only protect their own health but also set an example for patients and the community at large. During the 2022-2023 influenza season in the United States, 75.9% of HCPs reported receiving influenza vaccinations. Physicians (94.5%) and HCPs working in hospitals (85.7%) had the highest influenza vaccination coverage [2]. However, a recent meta-analysis of cross-sectional studies reporting seasonal influenza vaccination rates among HCPs from 26 nations across seven different regions reported a relatively low global influenza vaccination coverage rate of 41.7% [3]. The influenza vaccination coverage rate among HCPs varies widely across regions worldwide. After the United States, countries in the Middle East, Oceania, and Europe also have relatively high rates, although some face challenges such as vaccine shortages, low public awareness, and religious objections [3]. The lowest rate has been observed in Africa, where many countries lack the resources, infrastructure, and personnel to provide adequate vaccination services [3].

The Middle East and North Africa (ME' NA) region comprises both the poorest and richest countries in the world. However, data on influenza vaccination coverage rates are scarce. A 2010 study of influenza vaccination rates in the Middle East revealed that uptake was below the recommended levels and varied greatly from one country to another, from 24.7% in the United Arab Emirates to 67% in Kuwait [4]. Another study in Saudi Arabia reported a vaccination rate of 67.6% among HCPs in major hospitals [5]. While these studies provide piecemeal evidence for the motors and barriers behind HCP vaccination, to date, there have been no comparative studies of uptake among different Middle Eastern, Eurasian, and African countries.

Nevertheless, cross-country comparative studies are useful for several reasons. First, they provide a snapshot of attitudes toward vaccination at a point in time and allow comparisons across countries to identify “bright spots” countries, highlighting examples of good practices that may not be apparent when studied in isolation. They can also help policymakers identify policies and interventions that are most likely to impact uptake. For example, a recent comparative study across six European countries revealed that non-vaccinated HCPs were more likely to be characterized by an “hesitant” sentiment toward vaccination showcasing “a neutral, slightly negative, view of the importance of the influenza vaccine and a mitigated view of its impact (…) [as well as] weak feelings of knowledge and no clear sense of autonomy (intrinsic motivation).” However, the extent to which HCPs were associated with this motivational profile varied across countries, suggesting different paths for tailoring interventions in line with the underlying motors of vaccine uptake specific to a country [6].

Factors that influence the vaccination rate include the level of economic development, availability and affordability of vaccines, quality of healthcare systems, knowledge and attitudes of HCPs and the public toward influenza and its prevention, and cultural and social norms regarding health and vaccination [3]. However, HCPs’ support for vaccination can help dispel misconceptions and encourage higher vaccination rates, ultimately leading to a healthier population and reduction in the burden of influenza. This study aimed to explore the barriers to and drivers of HCPs vaccine acceptance and advocacy in countries in the Middle East, Eurasia, and Africa.

## Materials and methods

### Participants

The participants were HCPs from 10 countries in the ME'NA region: Azerbaijan, Egypt, Libya, Morocco, Nigeria, Pakistan, Russia, Saudi Arabia, Tunisia, and Türkiye. All HCPs (medical/paramedical) currently practicing medicine were eligible for inclusion.

### Design and procedure

We used surveys conducted in English, Arabic, French, Russian, Azerbaijani, and Turkish languages. While some countries prefer to disseminate surveys only in English (Saudi Arabia, Nigeria, and Pakistan), others prefer to use surveys in their official language (Türkiye, Azerbaijan, and Russia). Egypt and Libya conducted surveys in Arabic and English; Tunisia in Arabic and French; and Morocco in French. To validate the surveys in different languages, all teams were provided with a translation form that they completed by adding a translation of each item of the survey in the other language. They were then provided with a back-translation form presenting the items in another language and requiring their translation back into English by a different translator who did not have access to the English source text. Back-translation reviews were then shared with the teams, asking them to compare the back-translations with the original text, identify discrepancies, and discuss any changes that needed to be made in the final translated version. Thereafter, validated surveys in languages other than English were distributed across countries. The English version of the survey is provided in the supplemental documents.

Data were collected between 20^th^ December 2022, and 1^st^ March 2023. Participants voluntarily completed the survey via either an online or paper-based version. The data were screened for outliers on both motors of influenza vaccination acceptance (MoVac-Flu) and engagement with vaccination advocacy (MovAd). Cases with missing values or those flagged as multivariate outliers based on Mahalanobis distances were excluded from the analysis because this is an indication of a careless response [7].

The final sample included data from respondents who were recruited online by sharing a survey link with social media groups whose membership was composed of healthcare workers, mailing lists of healthcare professional associations, workshops attended by healthcare professionals, faculty alumni, and personal phone contacts.

Participants in Azerbaijan were recruited by posting a survey link to staff mailing lists of Ege Hospital, HB Guven Hospital, Central Customs Hospital, Bakı Sağlamlıq Mərkəzi, Baku Medical Plaza, Live Bona Dea Hospital, Azərbaycan Tibb Universiteti Tədris Cərrahiyə Klinikası, and Tədris Terapevtik Klinika. Participants in Egypt were recruited via Facebook groups (e.g., Alexandria Faculty of Medicine Graduates, Scientific research, Pediatric critical care), employees' WhatsApp Groups (Salah Al Awaidy Hospital, Amerya Hospital), and the Public Health of the Arab World mailing list. Participants from Libya, Morocco, Pakistan, and Türkiye were recruited using WhatsApp. Participants in Nigeria were recruited through WhatsApp groups, Facebook and Twitter accounts, professional and healthcare workers’ associations, and personal phone contacts. Faculty alumni were recruited as distributors to share their professional networks. Participants in Russia were recruited from among members of the Russian Scientific Medical Society of Internal Medicine, employees of the Russian National Research Medical University, and Moscow Polyclinic. Participants in Saudi Arabia were invited to participate via WhatsApp and Telegram groups as well as during workshops and seminars.

### Ethical considerations

All procedures were performed in compliance with relevant laws and institutional guidelines and approved by the institutional ethic committees of each country (on 25.10.2022 from DGK TXI BEK with protocol no: 01/22 in Azerbaijan; on 27.09.2022 from High Institute of Public Health-Alexandria University in Egypt; on 13.12.2022 from Benghazi Medical Center with decision no: 132.44.1 in Libya; on 20.02.2023 from Biomedical Research Ethics Committee of Mohammed V University–Rabat with decision no: 10/23 in Morrocco; on 25.10.2022 from King Fahad Medical City with decision no: 22-464E in Saudi Arabia; on 02.09.2022 from Abderrahmane Mami Hospital with approval no: 17/2022 in Tunisia; and on 18.10.2022 from Hacettepe University Non-Invasive Clinical Research Ethics Committee with decision no: 2022/16-50 in Türkiye). In Nigeria, Pakistan, and Russia, there are no legal or policy requirements for research involving healthcare or social care staff recruited as research participants by virtue of their professional roles.

The survey was designed in compliance with the principles outlined in the British Psychological Society Code of Ethics and included a consent form informing participants of their rights, validating their autonomy to participate and withdraw at any stage during the survey, and reiterating the anonymous nature of their participation in the study and the data they would provide.

### Measures

#### Motors of influenza vaccination acceptance (MoVac-Flu)

The 12-item MoVac-flu scale [8] measures the following sentiments: the sentiment that influenza vaccination is important, the sentiment that it is impactful, the feeling of knowing how the influenza vaccination works, and the sentiment of autonomy regarding influenza vaccination decisions. Vaccine acceptance sentiments are measured on a 7-point scale (1 = strongly disagree, 4 = neither disagree nor agree, 7 = strongly agree) to measure the participants’ thoughts about influenza vaccination (Cohen's α = 0.973).

#### Motors of engagement with vaccination advocacy (MovAd)

The 12-item MovAd scale [8] measures the following sentiments: the sentiment that vaccination advocacy is important; the sentiment that it is impactful; the feeling of knowing how to advocate vaccination; and the sentiment of autonomy in the decision to advocate vaccination. Vaccine advocacy sentiments were measured using the same 7-point Likert scale (Cohen's α = 0.902).

#### Behavioral measures

Behavioral measures included measures of advocacy behavior, where participants were asked how often they recommended the influenza vaccination to their colleagues, as well as self-vaccination behavior (by asking how often they had been vaccinated against influenza themselves, how comfortable they felt in getting the influenza vaccine themselves, whether they had been vaccinated in the 2021/2022 season [autumn/winter]), and if so, against what infection(s) they protected themselves against (influenza, tetanus, diphtheria, pertussis, COVID-19, or others).

#### Exploratory measures

Additional exploratory measures included asking participants how comfortable they were with receiving the influenza vaccine themselves, how often their colleagues received the influenza vaccine, and how difficult it was for them to incorporate influenza vaccination into their practice. They were also asked to report how often they recommended influenza vaccination to eligible patients (advocacy behavior).

### Sample size

Participants were recruited using a nonrandom snowball sampling method. Recent recommendations based on observational studies [9] have suggested a sample size of 500 to estimate parameters in a population and a minimum sample size of 200 (100 + 50 × 2) for a model with two independent variables (e.g., MoVac score and HCPs’ category). Therefore, the target sample size was 500 with an initial minimum target of 250 HCPs per professional category (medical practitioners, nurses, and pharmacists).

### Preliminary analyses

Descriptive statistics and alpha coefficients for the MoVac-Flu and MovAd scales are reported in Supplemental Table S1a, and the correlations are reported in Supplemental Table S1b. Normality assumptions were met as all kurtosis and skewness scores were below the upper threshold of 3.29 for large samples [10]. Cronbach's alpha for the autonomy subscale of MovAd-flu was less than 0.70, indicating some inconsistency in the responses to the autonomy items. However, the inter-item correlation between these items was 0.36, suggesting that the responses were moderately correlated with each other. Therefore, they were added together in a composite score in subsequent analyses.

The R package NbClust, version 3.0.1 [11] was used to determine the optimal number of clusters in the dataset. For the MoVac-flu subscale, the mean scores for importance, impact, knowledge, and autonomy were analyzed using the NbClust() function to calculate the best clustering solution. The optimal number of clusters was 2, with a silhouette value of 0.61, the highest compared to the other cluster solutions (0.52, 0.51, 0.48, and 0.44, for 3, 4, 5, and 6 clusters, respectively), and suggesting a “good” clustering solution. For the MovAd-flu subscale, the mean scores for importance, impact, and knowledge were analyzed. The optimal number of clusters for was two, with a silhouette value of 0.43, which was the highest compared to the other cluster solutions (0.32, 0.27, 0.28, and 0.27 for 3, 4, 5, and 6 clusters, respectively). This suggested a “fair” clustering solution.

One-way multivariate analysis of variance (MANOVA) was performed to compare MoVac-Flu and MoVac-Flu predictors (importance, impact, knowledge, and autonomy) across the two clusters.

## Results

A total of 872 HCPs (mainly medical practitioners) voluntarily took part in a questionnaire survey in Russia (n = 283), Morocco (n = 224), Türkiye (n = 95), Nigeria (n = 67), Pakistan (n = 60), Azerbaijan (n = 55), Tunisia (n = 55), Saudi Arabia (n = 16), Egypt (n = 7), and Libya (n = 7). After screening for outliers, the sample size was reduced to 721. Table 1 summarizes the demographic data including participants’ age, sex, and professional category (medical practitioners, nurses, and pharmacists) according to the countries.

**Table 1:** Demographic characteristics of the participants.

Variables	AZE	EGY	LBY	MAR	NGA	PAK	RUS	SAU	TUN	TUR	TOTAL
**N**	52	8	6	183	24	21	266	16	52	93	721
**Age, year Mean ± SD (min-max)**	35.3 ± 8.4 (21-62)	29.0 ± 4.2 (24-36)	47.7 ± 7.5 (34-55)	47.6 ± 9.9 (28-80)	31.1 ± 11.0 (20-61)	32.8 ± 10.1 (21-62)	42.4 ± 12.5 (23-75)	47.4 ± 10.1 (30-67)	51.5 ± 11.4 (28-72)	35.5 ± 10.1 (22-60)	42.3 ± 12.3 (20-80)
**Age groups**											
18-29 years, n (%)	16 (30.8)	5 (62.5)	0 (0.0)	9 (4.9)	16 (66.7)	13 (61.9)	60 (22.6)	1 (6.3)	1 (1.9)	41 (44.1)	162 (22.5)
30-49 years, n (%)	34 (65.4)	3 (37.5)	4 (66.7)	106 (57.9)	6 (25.0)	6 (28.6)	134 (50.4)	6 (37.5)	23 (44.2)	37 (39.8)	359 (49.8)
50-65 years, n (%)	2 (3.9)	0 (0.0)	2 (33.3)	62 (33.9)	2 (8.3)	2 (9.5)	65 (24.4)	8 (50.0)	23 (44.2)	15 (16.1)	181 (25.1)
Over 65 years, n (%)	0 (0.0)	0 (0.0)	0 (0.0)	6 (3.3)	0 (0.0)	0 (0.0)	7 (2.6)	1 (6.3)	5 (9.6)	0 (0.0)	19 (2.6)
**Sex, n (%)**											
Female	32 (61.5)	2 (25.0)	3 (50.0)	127 (69.4)	14 (58.3)	7 (33.3)	209 (78.6)	8 (50.0)	28 (53.8)	68 (73.1)	498 (69.1)
Male	19 (36.5)	6 (75.0)	3 (50.0)	55 (30.1)	10 (41.7)	14 (66.7)	56 (21.0)	7 (43.8)	24 (46.2)	25 (26.9)	219 (30.4)
Missing	1 (1.9)	0 (0.0)	0 (0.0)	1 (0.5)	0 (0.0)	0 (0.0)	1 (0.4)	1 (6.2)	0 (0.0)	0 (0.0)	4 (0.5)
**Profession, n (%)**											
Nurse/midwife	18 (34.6)	0 (0.0)	0 (0.0)	9 (4.9)	4 (16.7)	1 (4.8)	23 (8.6)	0 (0.0)	1 (1.9)	18 (19.4)	74 (10.3)
Pharmacist	0 (0.0)	1 (12.5)	0 (0.0)	10 (5.5)	4 (16.7)	3 (14.3)	1 (0.4)	0 (0.0)	2 (3.8)	26 (28.0)	47 (6.5)
Medical practitioner	34 (65.4)	7 (87.5)	6 (100.0)	164 (89.6)	16 (66.7)	17 (81.0)	242 (91.0)	16 (100.0)	49 (94.2)	49 (52.7)	600 (83.2)
**Practice setting, n (%)**											
Private practice	1 (1.9)	2 (25.0)	1 (16.7)	68 (37.2)	2 (8.3)	10 (47.6)	22 (8.3)	1 (6.3)	3 (5.8)	1 (1.1)	111 (15.4)
Group practice	1 (1.9)	0 (0.0)	0 (0.0)	2 (1.1)	2 (8.3)	1 (4.8)	29 (10.9)	1 (6.3)	0 (0.0)	8 (8.6)	44 (6.1)
Hospital employment	38 (73.1)	5 (62.5)	4 (66.7)	96 (52.5)	10 (41.7)	7 (33.3)	49 (18.4)	7 (43.8)	44 (84.6)	56 (60.2)	316 (43.8)
Locum tenens	0 (0.0)	0 (0.0)	0 (0.0)	0 (0.0)	1 (4.2)	0 (0.0)	54 (20.3)	0 (0.0)	0 (0.0)	0 (0.0)	55 (7.6)
Pharmacy	0 (0.0)	0 (0.0)	0 (0.0)	2 (1.1)	2 (8.3)	0 (0.0)	0 (0.0)	0 (0.0)	0 (0.0)	10 (10.8)	14 (1.9)
Other	12 (23.1)	1 (12.5)	1 (16.7)	15 (8.2)	7 (29.2)	3 (14.3)	112 (42.1)	7 (43.8)	5 (9.6)	18 (19.4)	181 (25.1)

AZE, Azerbaijan; EGY, Egypt; LBY, Libya; MAR, Morocco; NGA, Nigeria; PAK, Pakistan; RUS, Russia; SAU, Saudi Arabia; TUN, Tunisia; TUR, Türkiye

### MoVac-Flu

**Figure 1:**
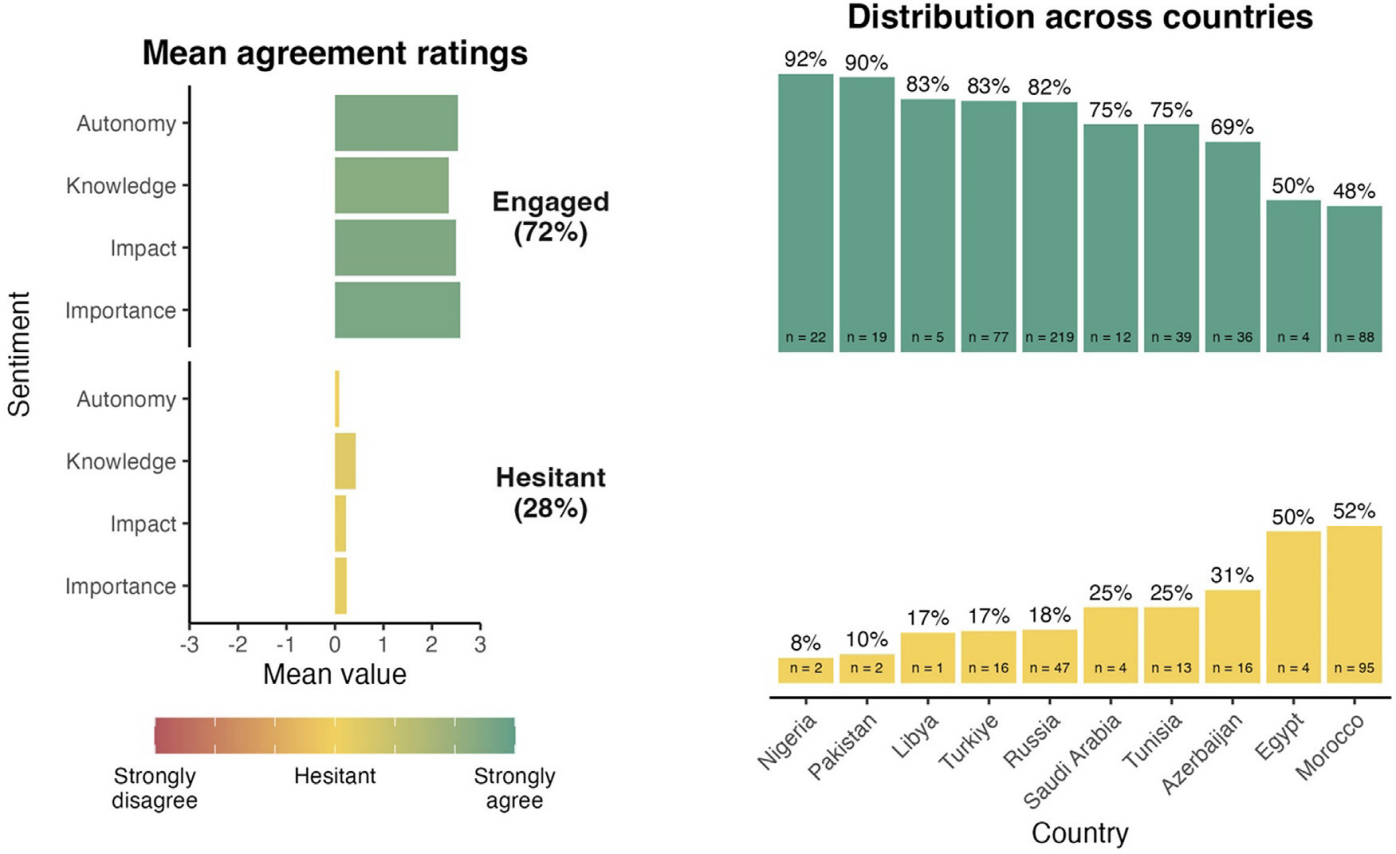
MoVac-flu profiles for the “engaged” and “hesitant” clusters and their distributions across countries ordered from the most to the least represented in the “engaged” cluster. *All countries had fewer than 100 respondents apart from Russia and Morocco; therefore, it is not possible to draw reliable conclusions from the rankings other than noting the larger size of the hesitant cluster in Morocco.

Figure 1 presents the mean agreement ratings for each predictor as a function of cluster membership and distribution of respondents across countries. The first sentiment cluster profile is the largest (N = 521, 72%). It is characterized by a strong sense that the influenza vaccine is important and impactful, a strong feeling of knowledge regarding the vaccine, and a strong sense of autonomy regarding the decision to be vaccinated. This sentiment profile was labeled “engaged.” By contrast, the second sentiment cluster is characterized by a neutral view of all dimensions. It was labeled “hesitant.”

The results of MANOVA, which was performed to compare the MoVac-flu predictors across the two clusters, showed a significant effect of the cluster on the dependent variables, thus confirming that all four dimensions of sentiments toward the influenza vaccination were highly differentiated between clusters (Wilks’ λ = 0.714, F[4, 716] = 446.63, *P* <0.001).

**Figure 2:**
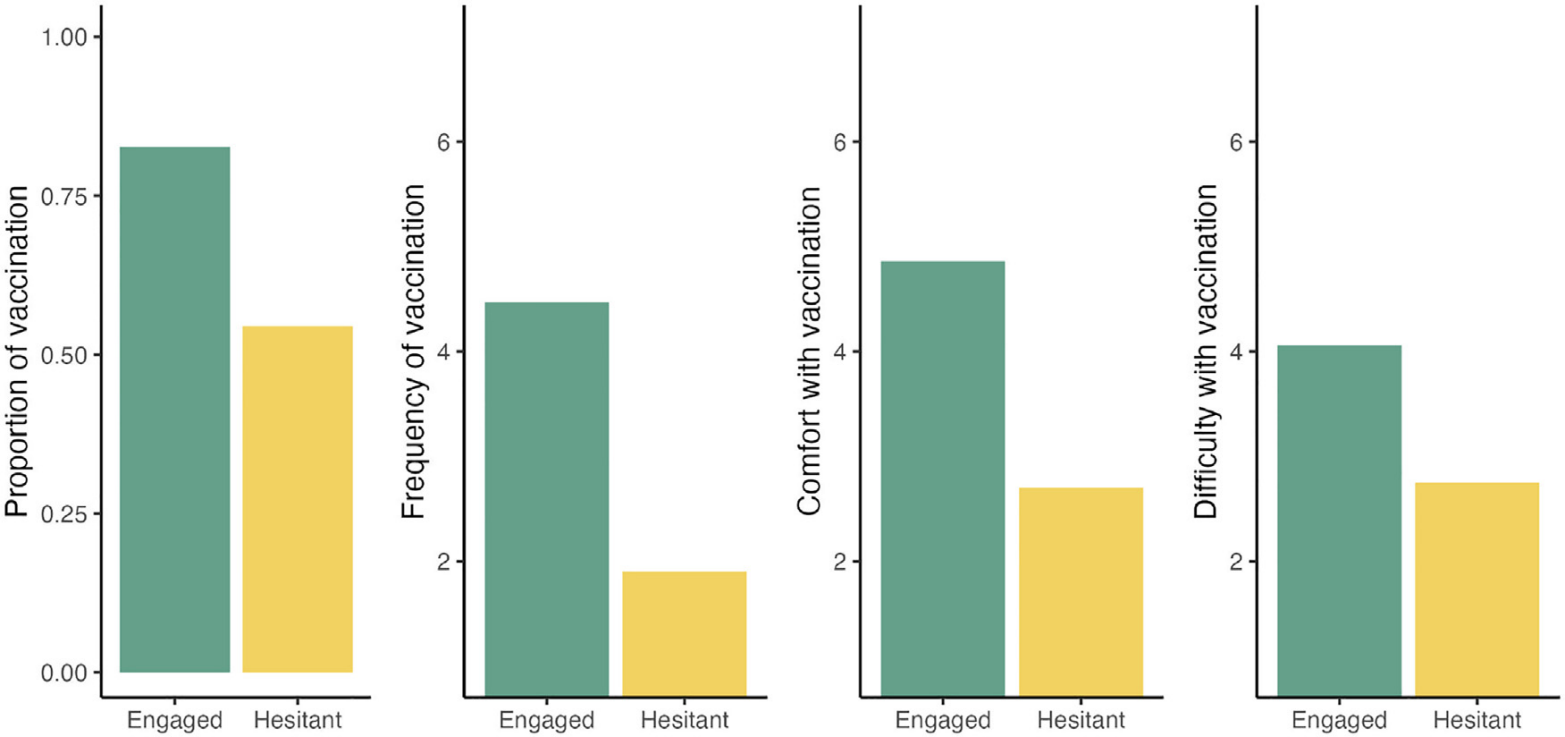
Proportion of respondents reporting getting vaccinated against the flu (panel 1), average frequency of getting the flu vaccine, from 1 = “Never” to 7 = “Always” (panel 2), comfort with getting vaccinated (panel 3), and difficulty with incorporating vaccination in their practice (panel 4) as a function of membership to the engaged vs hesitant sentiment cluster.

**Figure 3:**
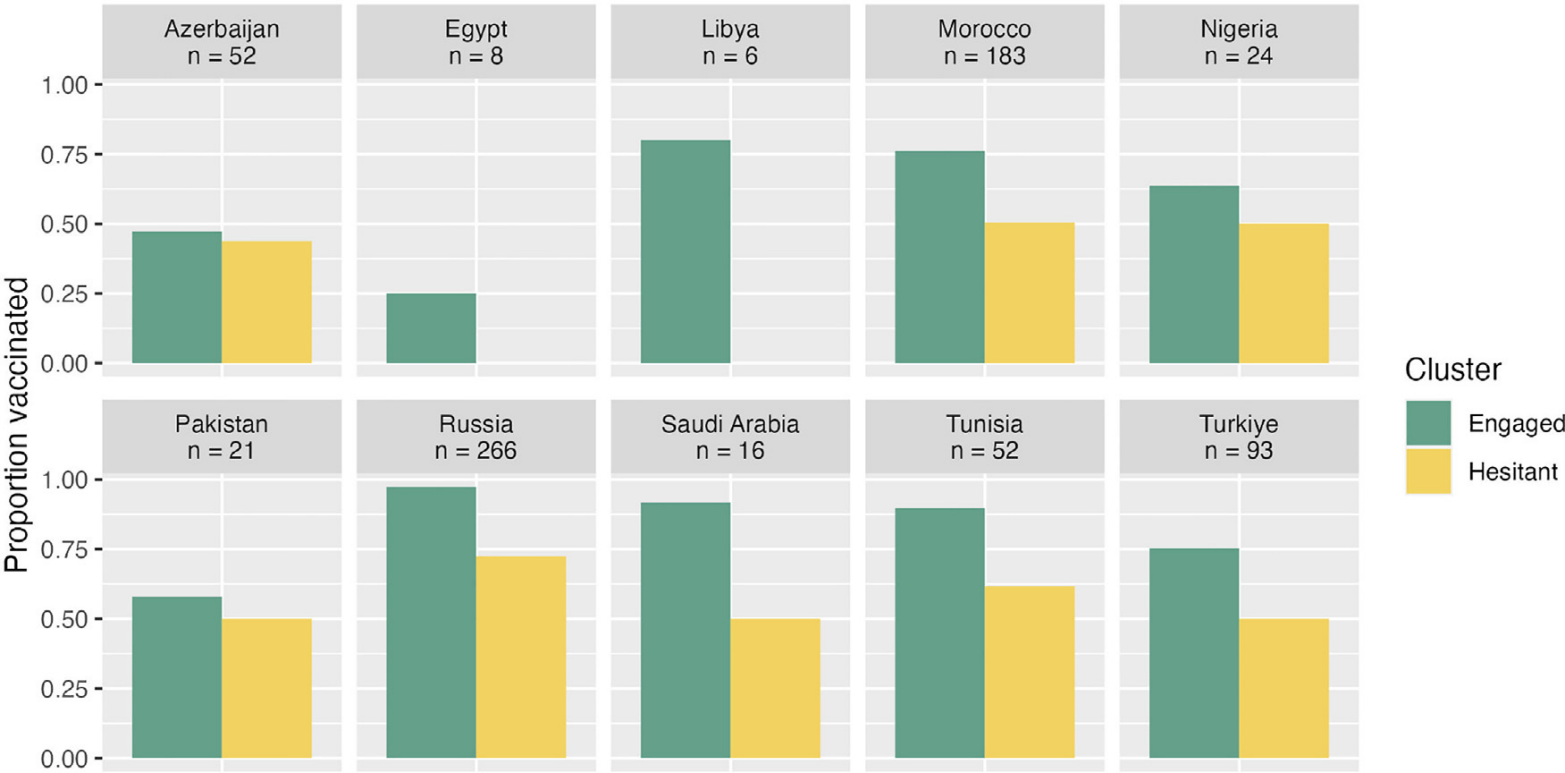
Proportion of respondents reporting getting vaccinated against the flu in each of the countries surveyed. *All countries had less than 100 respondents apart from Russia and Morocco, so it is not possible to draw reliable conclusions from the observed differences, other than noting that the association between membership and the engaged sentiment and the likelihood of being vaccinated remains positive in most countries.

**Figure 4:**
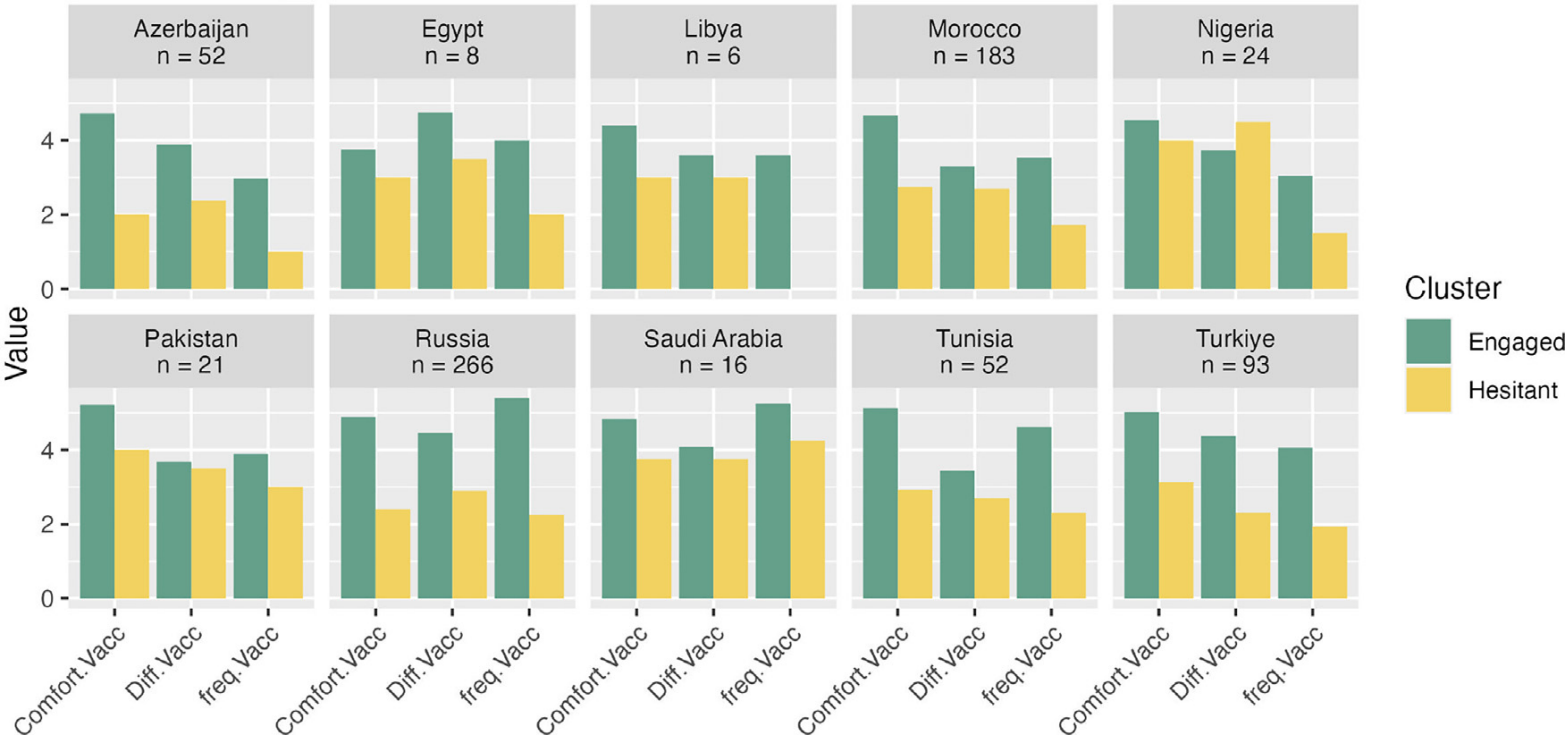
Average ratings for comfort with getting vaccinated, difficulty with incorporating vaccination in one's practice, and average frequency of getting the flu vaccine, from 1 to 7 as a function of vaccination sentiment cluster in each country sampled. *The same caveat regarding the sample size applies to these results.

The membership to the “engaged” sentiment cluster was examined to see if it was a predictor of self-vaccination behaviors. While 82.7% of the respondents in the engaged cluster reported having had the influenza vaccine in the past influenza season, only 54.5% did so in the hesitant cluster (a statistically significant gap, χ2[1, N = 721] = 59.76, *P* <0.001, see Figure 2, panel 1). On average, respondents in the engaged sentiments cluster reported being vaccinated against influenza often (MEngaged = 4.47, SD = 1.80), whereas respondents in the hesitant cluster reported being vaccinated against influenza extremely rarely (MHesitant = 1.90, SD = 1.88). This difference was statistically significant (Welch's t [347] = 16.7, *P* <0.001; Figure 2, panel 2). A similar pattern of results emerged when comparing responses to the question, “How comfortable are you with getting the flu vaccine yourself?” (Welch's t [275] = 17.7, *P* <0.001, see Figure 2, panel 3). How difficult is it for you to incorporate flu vaccination into your practice procedures?” (Welch's t [388] = 11.7, *P* <0.001; see Figure 2, panel 4). Finally, Figure 3, Figure 4 report these patterns within each country sample, with the caveat that the differences observed may be random variations owing to the small sample sizes.

### MovAd-Flu

**Figure 5:**
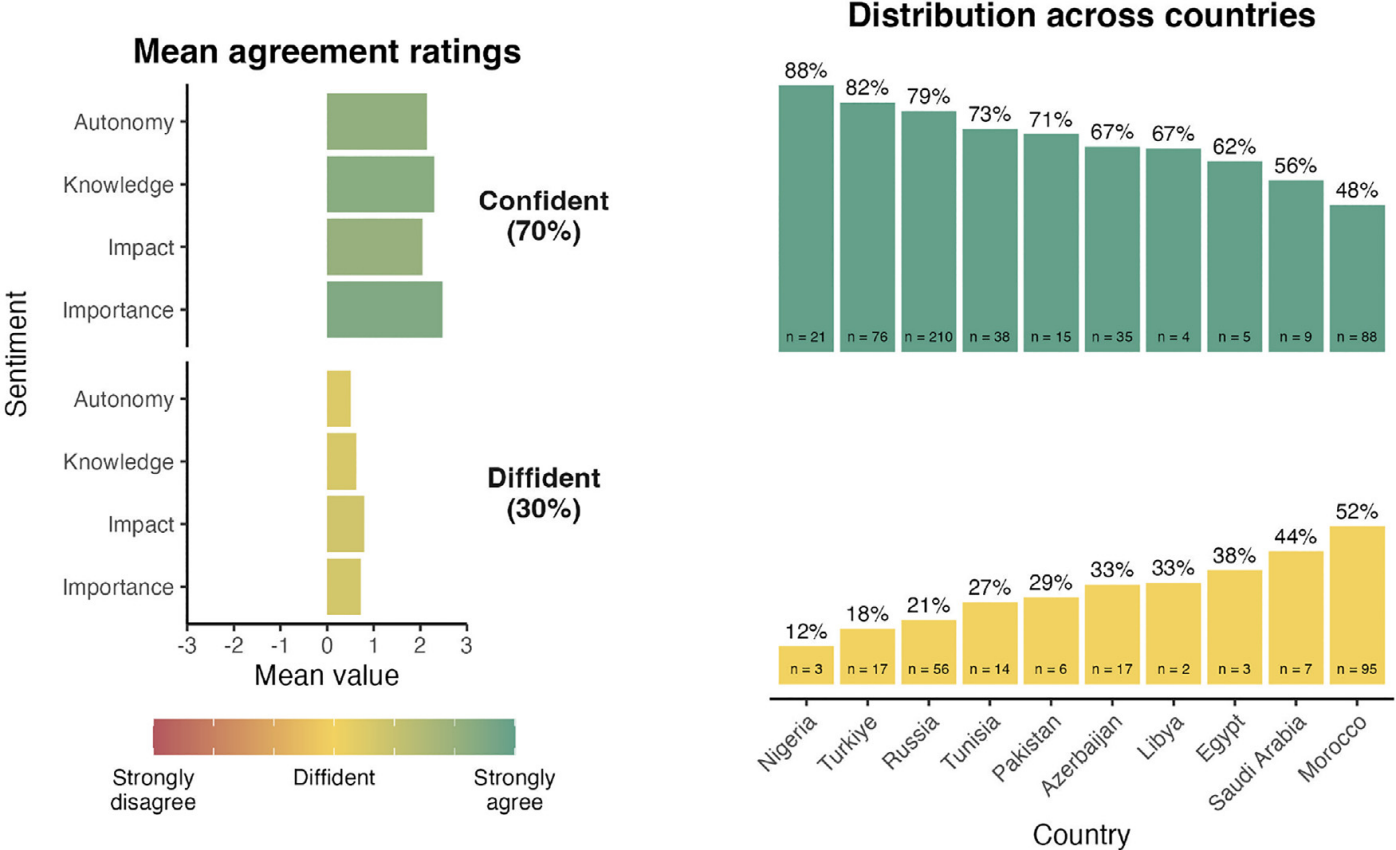
MovAd-flu profiles for the “confident” and “diffident” clusters and their distributions across countries ordered from the most to the least represented in the “confident” cluster.

Figure 5 illustrates the two cluster profiles and the distribution of respondents across countries. The first sentiment cluster profile is the largest (N = 501; 70%). It is characterized by a positive sense that advocacy for the IV is important and a positive feeling of knowledge and autonomy regarding advocacy practices. The feeling of impact (the effectiveness of advocacy) was slightly less positive. This sentiment profile was labeled “confident.” By contrast, the second sentiment cluster is characterized by a neutral view of all dimensions. It was labeled “diffident” to represent a more unassuming sentiment toward advocacy.

According to the MANOVA performed to compare the MoVac-flu predictors across the two clusters, a significant effect of the cluster on the dependent variables was determined, thus confirming that all four dimensions of sentiments toward advocacy for influenza vaccination were highly differentiated between clusters (Wilks’ λ = 0.652, F [4, 716] = 335.91, *P* <0.001).

**Figure 6:**
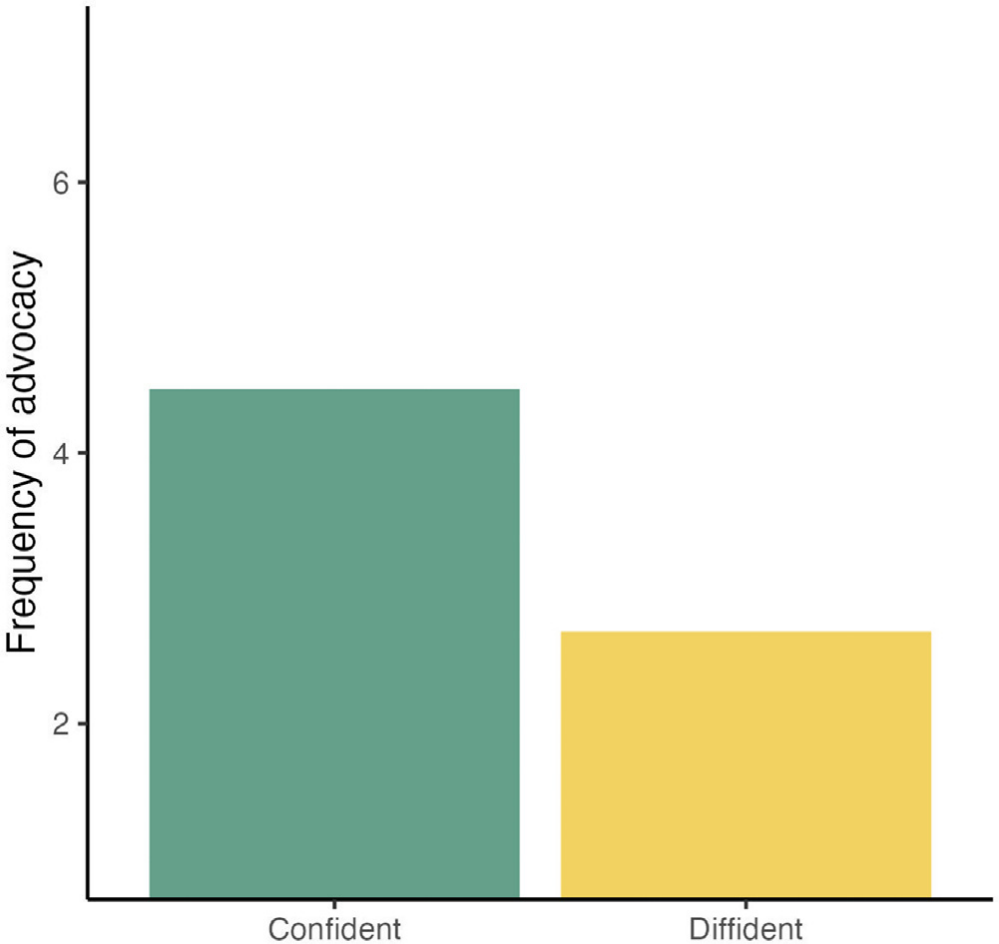
Average frequency of recommending flu vaccination to colleagues, from 1 = “Never” to 7 = “Always” as a function of membership to the confident vs diffident sentiment cluster.

**Figure 7:**
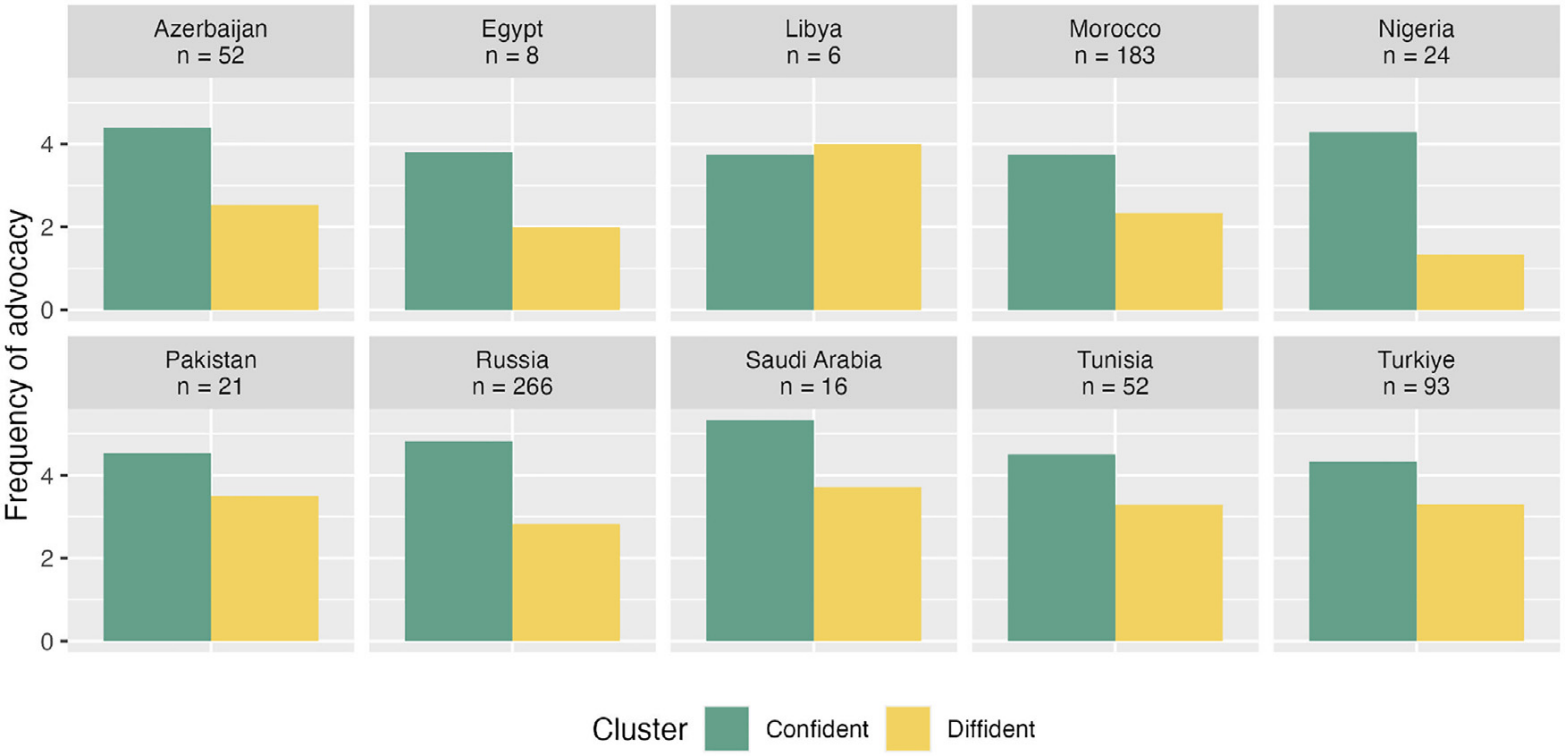
Average ratings for the frequency of recommending flu vaccination to colleagues as a function of advocacy sentiment cluster in each country sampled.

**Figure 8:**
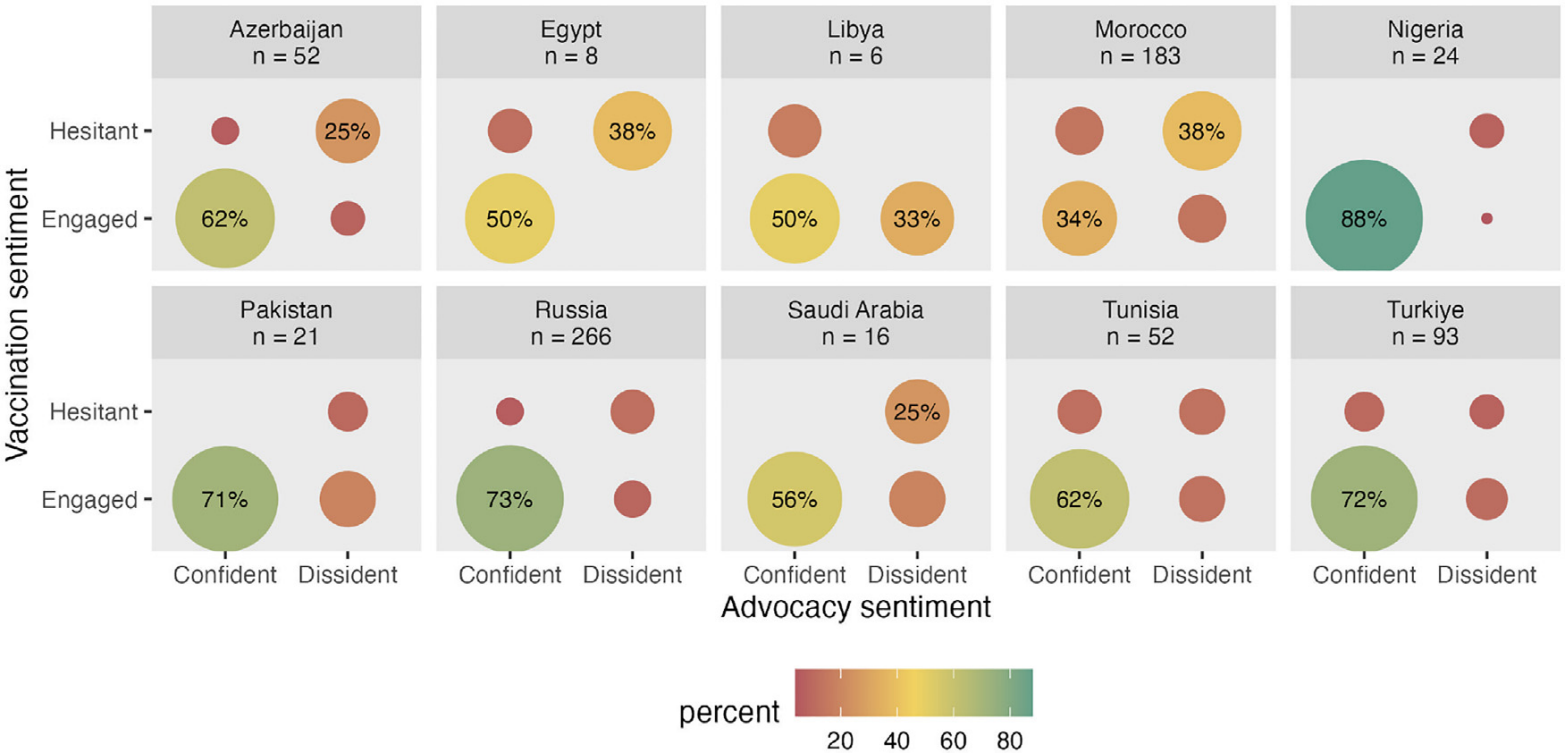
Proportion of healthcare providers by vaccination and advocacy cluster membership. *The dots without labels represented less than 20% (1 in 5) of the sample collected in each country.

The membership to the “confident” sentiment cluster was examined if it was a predictor of advocacy behavior, namely the extent to which participants reported recommending the influenza vaccination to their colleagues. On average, respondents in the confident sentiment cluster often advocated influenza vaccination for their colleagues (MEngaged = 4.47, SD = 1.43), whereas respondents in the diffident cluster reported that they rarely engaged in this type of behavior (MDiffident = 2.68, SD = 1.57). This difference was statistically significant (Welch's t [385] = 14.43, *P* <0.001; Figure 6). Figure 7 shows these patterns within each country sample, with the caveat that the differences observed may be random variations owing to the small sample sizes in some countries. Finally, Figure 8 shows the proportion of HCPs in each combination of sentiment clusters. This suggests that there may be differences between countries. For example, most HCPs surveyed in Russia felt engaged in influenza vaccination and were confident in recommending influenza vaccination to their colleagues. In contrast, about two out of five HCPs surveyed in Morocco felt hesitant about influenza vaccination and dissident about recommending vaccination to colleagues.

## Discussion

This study provides an initial overview of the motors and barriers faced by HCPs working in the Middle East, Eurasia, and Africa regions, with regards to getting vaccinated against influenza themselves, and with regards to advocating influenza vaccination to their colleagues. According to the results we obtained, the majority of HCPs who took part in the study felt positively engaged with influenza vaccination, showcasing strong beliefs about the importance of influenza vaccination and its effectiveness, as well as a strong sentiment of autonomy to get vaccinated, suggesting that influenza vaccination was a self-regulated behavior in this cluster. However, the analysis also revealed the existence of a smaller cluster of HCPs who were more hesitant about influenza vaccination. Hesitant sentiments are characterized by uncertain views about the importance, impact, knowledge, and autonomy of influenza vaccination.

While every effort was made to disseminate the survey widely, data collection relied on opportunity sampling and payment-in-kind from the HCPs in each country, who donated their time to oversee ethics and data collection. Hence, this study did not epidemiologically represent each country. Moreover, the resulting small sample sizes in many of the participating countries meant that it was not possible to draw conclusions about the differences between all countries and different groups of HCPs. However, there was a notable difference between Morocco and Russia (where the samples were sufficiently large to allow for a tentative comparison). The respondents in Morocco were more likely to show hesitant sentiments than the HCPs surveyed in Russia (52% vs 18%). However, without further exploration, it is difficult to assess the cause of this difference, which could be due to differences in sampling bias, desirability bias, or systemic differences between the two countries. In contrast, the results gave us the opportunity to analyze the whole dataset in terms of the relationship of each sentiment cluster with vaccination behaviors of HCPs in general. Future research should benefit from partnerships with local hospitals to allow for a truly random selection of HCPs to prevent sampling bias. It would also benefit from independent research funding to support the hiring of local researchers who could assist in the management of the data collection in the different countries.

Cluster membership was a strong predictor of overall vaccination behavior and attitudes. Those who were classified as showing an engaged sentiment toward influenza vaccination were also more likely to be vaccinated themselves, to have been frequently vaccinated in the past, to feel comfortable being vaccinated themselves, and to feel that it was easier to implement the influenza vaccination in their practice compared to those who were characterized by a hesitant MoVac-flu sentiment. This latter finding may suggest that perceived difficulty in implementing influenza vaccination could be a barrier; however, since the HCPs in this sample also reported being uncertain about the value of influenza vaccination, it is plausible that the perceived difficulty of implementation in practice is a consequence rather than a cause of their hesitant sentiment.

A similar pattern was observed when analyzing data on influenza vaccination advocacy among HCPs, where two natural clusters were identified within the data: a cluster of higher scores on all four dimensions of the MovAd-flu scale, labeled “confident” and a cluster characterized by lower scores throughout, labeled “diffident.” The second cluster was also smaller in size, suggesting that the majority of the surveyed HCPs felt confident about advocating vaccination. However, these two distinct sentiments were related to the frequency with which people reported recommending influenza vaccination to their colleagues. Those who felt differently about advocacy also reported recommending vaccination much less often.

Several potential packages of interventions can be inspired by these results to encourage hesitant HCP to self-vaccinate and to support vaccination advocacy. First, to encourage influenza vaccination among HCPs, strategies that focus on communication campaigns tailored to minority groups characterized by hesitant sentiments toward influenza vaccination could be efficient. For example, past research [12] has shown that autonomy-supportive communication can improve the effectiveness of statements seeking to promote HCPs’ influenza vaccination uptake, especially for those who tend to see influenza vaccination as unimportant and unconnected with their internal values or tend to be unwilling to act to get vaccinated. Examples of autonomy-supportive messages include “By choosing to protect ourselves against the virus, we'll reduce our risk of developing flu-related health complications,” compared to more controlling styles of communication, such as “You must protect yourself against the virus to reduce your risk of developing flu-related health complications.” This could be combined with a change in the environment, aiming to make vaccination easier and more convenient. For example, setting up vaccination clinics onsite, near the workplace, or through mobile carts would enable HCPs to easily access influenza vaccines. The clinics were available during convenient hours, such as before or after shifts, and HCPs were reminded about the clinics via email, posters, and autonomy-supportive communication [13]. Another approach would be to highlight the relevant social norms to encourage hesitant HCPs to be vaccinated. Research has shown that social norms work best when they relate to distinct and close social groups [14]. Therefore, it could be efficient to highlight that the percentage of HCPs who had not frequently been vaccinated in the past joined the group of those who were already vaccinated via posters or other forms of communication. It is important to be cautious and ensure that any message aimed at encouraging influenza vaccination among HCPs highlights that most of their colleagues have been vaccinated. These interventions can be further enhanced by implementing management or organizational changes. This may include assigning personnel dedicated to designing influenza vaccination uptake intervention programs tailored to employees, requiring active declination, or providing incentives such as extra time off or other forms of recognition [13].

The second set of interventions could focus on boosting advocacy for influenza vaccination among HCPs. Providing a sense of ownership by allowing HCPs to take the opportunity to choose the type or location of vaccination can, in turn, make them more likely to advocate influenza vaccination to their colleagues. An additional intervention could be to provide materials to support advocacy to those who are already engaged in vaccination and vaccination advocacy; for example, providing how-to guides highlighting communication strategies such as motivational interviewing techniques to advocate influenza vaccination to colleagues could contribute to making HCPs more confident. The use of visible signs of vaccination, such as stickers or colored lanyards, could be an alternative approach to encourage advocacy for those who feel less confident about influencing others through conversations [15].

In conclusion, sentiments toward influenza vaccination tended to be strongly associated with sentiments toward advocacy, such that those who felt engaged in influenza vaccination also felt confident about advocating and vice versa, with some initial evidence that the strength of this relationship may vary across countries. Culturally sensitive interventions to encourage influenza vaccination among hesitant HCPs and to boost advocacy activities for influenza vaccination among engaged HCPs could be an effective approach to increase influenza vaccination coverage among healthcare providers in the Middle East, Eurasia, and Africa.

## Funding

The Middle East, Eurasia, and Africa Influenza Stakeholders Network (ME'NA-ISN) received a fund for the execution and reporting of this study from Sanofi. The sponsor had no role in the methodology, data collection, analysis, or reporting of the study results. ME'NA-ISN was responsible for the scientific content and accuracy of the data. The funder has no editorial control over the reported work.

## Author contributions

**Mine Durusu Tanriover:** Conceptualization, project administration, validation, investigation, writing–original draft, writing–review and editing, and supervision. **Gaelle Vallee-Tourangeau:** Conceptualization, methodology, software, validation, investigation, formal analysis, data curation, writing - original draft. **Valentin A Kokorin:** Investigation, resources, data curation, writing - review, and editing. **Vera N Larina:** investigation, resources, and data curation. **Mouna Maamar:** Investigation, resources, data curation, writing - review & editing. **Hicham Harmouche:** Investigation, resources, data curation. **Oğuz Abdullah Uyaroglu:** Investigation, resources, data curation, writing - review & editing. **Dilan Yağmur Kutlay:** Investigation, resources, data curation. **Jalila Ben Khelil:** Investigation, resources, data curation, writing - review & editing. **Abdul-Azeez A Anjorin:** Investigation, resources, data curation, writing - review, and editing. **Muhammad Suleman Rana:** Investigation, resources, data curation, writing - review & editing. **Jabrayil Jabrayilov:** Investigation, resources, data curation, writing - review, and editing. **Fatima Al Slail:** Investigation, resources, data curation, writing - review & editing. **Dalal Al Kathiry:** Investigation, resources, data curation, writing -review and editing**. Hasina Al Harthi:** Investigation, resources, data curation, writing - review & editing. **Ramy Mohamed Ghazy:** Investigation, resources, data curation, writing - review and editing. **Milad Gahwagi:** Investigation, resources, data curation, writing - review & editing. **Alireza Mafi:** Conceptualization, Funding acquisition. **Parvaiz Koul:** Conceptualization, supervision, funding acquisition. **Salah Al Awaidy:** Conceptualization, supervision, funding acquisition. **All the authors** reviewed and approved the final version of the publication.

## Data availability

Data will be made available on request.

## Declarations of competing interest

Mine Durusu Tanriover, Gaelle Vallee-Tourangeau, Valentin A Kokorin, Mouna Maamar, Oğuz Abdullah Uyaroglu, Dilan Yağmur Kutlay, Jalila Ben Khelil, Abdul-Azeez A Anjorin, Muhammad Suleman Rana, Jabrayil Jabrayilov, Fatima Al Slail, Dalal XX, Hasina Al Harthi, Ramy Mohamed Ghazy, Milad Gahwagi, Parvaiz Koul, and Salah Al Awaidy have no conflict of interests related to this publication. Alireza Mafi is an employee of Sanofi Vaccines and holds company shares or stocks.

## Appendix Supplementary materials

Image, application 1

Image, application 2
