# Supplementary material for: The vaccination acceptance, confidence, and conviction on influenza in the Middle East, Eurasia, and Africa among healthcare providers (VACCIMENA-HCP) project 2023: Determinants of vaccination behavior
Source: IJID Reg. 2025 Jan 19;14:100572. doi: 10.1016/j.ijregi.2025.100572 (PMC11871459; doi:10.1016/j.ijregi.2025.100572)
Supplement: Supplementary file 1 [file mmc1.docx]

**Supplemental Materials**

**Table S1a.** Descriptive statistics and coefficient α for MoVac-flu and MovAd sentiments

| **Sentiment** | **M** | **SD** | **Skewness** | **Kurtosis** | **α** |
| --- | --- | --- | --- | --- | --- |
|  | **MoVac-flu sentiments** | | | | |
| Importance | 4.93 | 1.27 | -1.42 | 1.85 | 0.929 |
| Impact | 4.87 | 1.25 | -1.36 | 1.77 | 0.907 |
| Knowledge | 4.81 | 1.12 | -1.05 | 0.82 | 0.811 |
| Autonomy | 4.86 | 1.31 | -1.30 | 1.34 | 0.935 |
|  | **MovAd-flu sentiments** | | | | |
| Importance | 4.94 | 1.14 | -1.46 | 2.56 | 0.871 |
| Impact | 4.66 | 1.03 | -1.39 | 2.92 | 0.765 |
| Knowledge | 4.79 | 1.11 | -1.08 | 0.99 | 0.881 |
| Autonomy | 4.64 | 1.12 | -0.78 | 0.24 | 0.630^a^ |

M: Mean, MovAd: Motors of engagement with vaccination advocacy, MoVac-Flu: Motors of influenza vaccination acceptance, SD: Standard deviation

^a^ This value is below the accepted level of 0.70. This sub-scale was not used for the cluster analyses although values on each item was examined.

**Table S1b.** Correlation coefficients for MoVac-flu and MovAd sentiments

| **Sentiment** | **1** | **2** | **3** |
| --- | --- | --- | --- |
|  | **MoVac-flu sentiments** | | |
| 1. Importance |  |  |  |
| 1. Impact | 0.944 |  |  |
| 1. Knowledge | 0.845 | 0.855 |  |
| 1. Autonomy | 0.960 | 0.943 | 0.851 |
|  | **MovAd-flu sentiments** | | |
| 1. Importance |  |  |  |
| 1. Impact | 0.646 |  |  |
| 1. Knowledge | 0.623 | 0.529 |  |
| 1. Autonomy | 0.621 | 0.507 | 0.575 |

MovAd: Motors of engagement with vaccination advocacy, MoVac-Flu: Motors of influenza vaccination acceptance

All correlation coefficients were statistically significant at a p value <0.001.

Table S2. Demographic breakdown by Movac clusters

| **Variables** | **Category** | **Movac cluster** | AZE | EGY | LBY | MAR | NGA | PAK | RUS | SAU | TUN | TUR | TOTAL |
| --- | --- | --- | --- | --- | --- | --- | --- | --- | --- | --- | --- | --- | --- |
| Age Groups | (18,30] | Engaged | 12 (23.1) | 2 (25) |  | 7 (3.8) | 14 (58.3) | 11 (52.4) | 49 (18.4) |  | 1 (1.9) | 33 (35.5) | 129 (100) |
| Age Groups | (18,30] | Hesitant | 4 (7.7) | 3 (37.5) |  | 2 (1.1) | 2 (8.3) | 2 (9.5) | 11 (4.1) | 1 (6.2) |  | 8 (8.6) | 33 (100) |
| Age Groups | (30,50] | Engaged | 23 (44.2) | 2 (25) | 3 (50) | 42 (23) | 6 (25) | 6 (28.6) | 109 (41) | 4 (25) | 13 (25) | 31 (33.3) | 239 (100) |
| Age Groups | (30,50] | Hesitant | 11 (21.2) | 1 (12.5) | 1 (16.7) | 64 (35) |  |  | 25 (9.4) | 2 (12.5) | 10 (19.2) | 6 (6.5) | 120 (100) |
| Age Groups | (50,65] | Engaged | 1 (1.9) |  | 2 (33.3) | 36 (19.7) | 2 (8.3) | 2 (9.5) | 55 (20.7) | 7 (43.8) | 20 (38.5) | 13 (14) | 138 (100) |
| Age Groups | (50,65] | Hesitant | 1 (1.9) |  |  | 26 (14.2) |  |  | 10 (3.8) | 1 (6.2) | 3 (5.8) | 2 (2.2) | 43 (100) |
| Age Groups | (65,90] | Engaged |  |  |  | 3 (1.6) |  |  | 6 (2.3) | 1 (6.2) | 5 (9.6) |  | 15 (100) |
| Age Groups | (65,90] | Hesitant |  |  |  | 3 (1.6) |  |  | 1 (0.4) |  |  |  | 4 (100) |
| Sex | Female | Engaged | 21 (40.4) | 1 (12.5) | 2 (33.3) | 54 (29.5) | 13 (54.2) | 7 (33.3) | 167 (62.8) | 6 (37.5) | 19 (36.5) | 55 (59.1) | 345 (100) |
| Sex | Female | Hesitant | 11 (21.2) | 1 (12.5) | 1 (16.7) | 73 (39.9) | 1 (4.2) |  | 42 (15.8) | 2 (12.5) | 9 (17.3) | 13 (14) | 153 (100) |
| Sex | Male | Engaged | 15 (28.8) | 3 (37.5) | 3 (50) | 33 (18) | 9 (37.5) | 12 (57.1) | 51 (19.2) | 5 (31.2) | 20 (38.5) | 22 (23.7) | 173 (100) |
| Sex | Male | Hesitant | 4 (7.7) | 3 (37.5) |  | 22 (12) | 1 (4.2) | 2 (9.5) | 5 (1.9) | 2 (12.5) | 4 (7.7) | 3 (3.2) | 46 (100) |
| Sex | Other / Prefer not to say | Engaged |  |  |  | 1 (0.5) |  |  | 1 (0.4) | 1 (6.2) |  |  | 3 (100) |
| Sex | Other / Prefer not to say | Hesitant | 1 (1.9) |  |  |  |  |  |  |  |  |  | 1 (100) |
| Profession | Midwife | Engaged | 1 (1.9) |  |  |  |  |  |  |  |  |  | 1 (100) |
| Profession | Nurse | Engaged | 7 (13.5) |  |  | 9 (4.9) | 4 (16.7) | 1 (4.8) | 19 (7.1) |  |  | 13 (14) | 53 (100) |
| Profession | Nurse | Hesitant | 10 (19.2) |  |  |  |  |  | 4 (1.5) |  | 1 (1.9) | 5 (5.4) | 20 (100) |
| Profession | Pharmacist | Engaged |  | 1 (12.5) |  | 4 (2.2) | 4 (16.7) | 2 (9.5) | 1 (0.4) |  | 2 (3.8) | 22 (23.7) | 36 (100) |
| Profession | Pharmacist | Hesitant |  |  |  | 6 (3.3) |  | 1 (4.8) |  |  |  | 4 (4.3) | 11 (100) |
| Profession | Physician/medical practitioner | Engaged | 28 (53.8) | 3 (37.5) | 5 (83.3) | 75 (41) | 14 (58.3) | 16 (76.2) | 199 (74.8) | 12 (75) | 37 (71.2) | 42 (45.2) | 431 (100) |
| Profession | Physician/medical practitioner | Hesitant | 6 (11.5) | 4 (50) | 1 (16.7) | 89 (48.6) | 2 (8.3) | 1 (4.8) | 43 (16.2) | 4 (25) | 12 (23.1) | 7 (7.5) | 169 (100) |
| Practice Setting | Group practice | Engaged | 1 (1.9) |  |  |  | 2 (8.3) | 1 (4.8) | 25 (9.4) | 1 (6.2) |  | 7 (7.5) | 37 (100) |
| Practice Setting | Group practice | Hesitant |  |  |  | 2 (1.1) |  |  | 4 (1.5) |  |  | 1 (1.1) | 7 (100) |
| Practice Setting | Hospital employment | Engaged | 24 (46.2) | 1 (12.5) | 4 (66.7) | 46 (25.1) | 9 (37.5) | 7 (33.3) | 37 (13.9) | 4 (25) | 32 (61.5) | 45 (48.4) | 209 (100) |
| Practice Setting | Hospital employment | Hesitant | 14 (26.9) | 4 (50) |  | 50 (27.3) | 1 (4.2) |  | 12 (4.5) | 3 (18.8) | 12 (23.1) | 11 (11.8) | 107 (100) |
| Practice Setting | Locum tenens | Engaged |  |  |  |  | 1 (4.2) |  | 44 (16.5) |  |  |  | 45 (100) |
| Practice Setting | Locum tenens | Hesitant |  |  |  |  |  |  | 10 (3.8) |  |  |  | 10 (100) |
| Practice Setting | Other | Engaged | 10 (19.2) | 1 (12.5) |  | 7 (3.8) | 6 (25) | 3 (14.3) | 95 (35.7) | 6 (37.5) | 4 (7.7) | 16 (17.2) | 148 (100) |
| Practice Setting | Other | Hesitant | 2 (3.8) |  | 1 (16.7) | 8 (4.4) | 1 (4.2) |  | 17 (6.4) | 1 (6.2) | 1 (1.9) | 2 (2.2) | 33 (100) |
| Practice Setting | Pharmacy | Engaged |  |  |  | 1 (0.5) | 2 (8.3) |  |  |  |  | 8 (8.6) | 11 (100) |
| Practice Setting | Pharmacy | Hesitant |  |  |  | 1 (0.5) |  |  |  |  |  | 2 (2.2) | 3 (100) |
| Practice Setting | Private practice | Engaged | 1 (1.9) | 2 (25) | 1 (16.7) | 34 (18.6) | 2 (8.3) | 8 (38.1) | 18 (6.8) | 1 (6.2) | 3 (5.8) | 1 (1.1) | 71 (100) |
| Practice Setting | Private practice | Hesitant |  |  |  | 34 (18.6) |  | 2 (9.5) | 4 (1.5) |  |  |  | 40 (100) |

AZE: Azerbaijan, EGY: Egypt, LBY: Libya, MAR: Morocco, NGA: Nigeria, PAK: Pakistan, RUS: Russia, SAU: Saudi Arabia, TUN: Tunisia, TUR: Turkiye
