# Supplementary material for: The vaccination acceptance, confidence, and conviction on influenza in the Middle East, Eurasia, and Africa among healthcare providers (VACCIMENA-HCP) project 2023: Determinants of vaccination behavior
Source: IJID Reg. 2025 Jan 19;14:100572. doi: 10.1016/j.ijregi.2025.100572 (PMC11871459; doi:10.1016/j.ijregi.2025.100572)
Supplement: Supplementary file 2 [file mmc2.docx]

**MENA-ISN SURVEY 2022**

**Consent**

Please confirm you agree with all the consent statements by selecting 'I consent'.

□ I consent
□ I do not consent *(Skip to section 6 (Thank you for your participation!))*

**YOUR THOUGHTS ABOUT VACCINATION**

Please indicate how much you agree or disagree with each of the following statements by selecting a number on the adjacent scale:

*(Response options for each item: Completely disagree, Mostly disagree, Slightly disagree, Neutral, Slightly agree, Mostly agree, Completely agree)*

1. When I talk openly about the flu vaccination, it has a positive impact on my patients' beliefs.
2. The flu vaccination is an important topic I want to discuss with my patients.
3. My patients' opinions of the flu vaccination can really be influenced by the conversations I have with them.
4. Discussing the flu vaccination with my patients is entirely my choice.
5. It is important that I mention the topic of the flu vaccination to my patients.
6. It matters that I talk openly about the flu vaccination with my patients.
7. If I discuss the flu vaccination, it will very much change my patients' views on this topic.
8. I am confident I can answer questions that my patients could ask me about the flu vaccination.
9. It is up to me to decide whether to have conversations about the flu vaccination with my patients.
10. I feel able to discuss the flu vaccination.
11. I know exactly how to talk about the flu vaccination with my patients.
12. I personally believe it is important to talk about the flu vaccination with my patients.

**What do you think about the flu vaccination in general?**

1. Vaccination of healthcare workers is a very effective way to protect vulnerable patients against the flu.
2. I know very well how vaccination of healthcare workers protects patients from the flu.
3. It is important that healthcare workers get the flu vaccination.
4. I understand how the flu vaccination of healthcare workers protects their vulnerable patients from the influenza infection.
5. The contribution of healthcare workers' flu vaccination to their vulnerable patients' health and well-being is very important.
6. I've carefully thought about flu vaccination for healthcare workers and believe it's the right thing to do to protect their vulnerable patients.
7. Giving the flu vaccination to healthcare workers has a positive influence on their vulnerable patients' health.
8. The flu vaccination of healthcare workers plays an important role in protecting their vulnerable patients' life.
9. I personally believe healthcare workers who vaccinate against the flu are protecting their vulnerable patients' health.
10. Vaccination of healthcare workers greatly reduces their vulnerable patients' risk of getting a severe flu.
11. I am knowledgeable about the flu vaccine.
12. I personally believe it is important that healthcare workers get vaccinated against the flu.

**Practice and Experience**

1. How often do you recommend the flu vaccination to your colleagues?
   *(Never, Extremely rarely, Rarely, Sometimes, Often, Extremely often, Always)*
2. How often have you been vaccinated against the flu yourself?
   *(Never, Extremely rarely, Rarely, Sometimes, Often, Extremely often, Always)*
3. How often do your colleagues get the flu vaccine?
   *(Never, Extremely rarely, Rarely, Sometimes, Often, Extremely often, Always)*
4. How comfortable are you with getting the flu vaccine yourself?
   *(Extremely uncomfortable, Very uncomfortable, Slightly uncomfortable, Neutral, Slightly comfortable, Very comfortable, Extremely comfortable)*
5. How difficult is it for you to incorporate flu vaccination into your practice procedures?
   *(Extremely difficult, Very difficult, Slightly difficult, Neutral, Slightly easy, Very easy, Extremely easy)*
6. Did you get vaccinated during the last autumn/winter (09/2021 - 03/2022)?

□ Yes
□ No

1. If you answered yes at the previous question, what infection(s) did you protect yourself against? (Tick all that apply)

□ Influenza
□ Tetanus, diphtheria, pertussis
□ COVID-19
□ Other: ____________

1. What is the main reason that led you to decide to get (or not to get) vaccinated?
   [Open text response]

**Finally, we would like to know a little bit about you.**

1. What is your profession?

□ Physician/medical practitioner
□ Pharmacist
□ Nurse
□ Midwife
□ Other: ____________

1. Where do you practice? (Select all that apply)

□ Private practice
□ Group practice
□ Hospital employment
□ Locum tenens
□ Pharmacy
□ Other: ____________

1. In which country do you practice?
   [Open text response]
2. What gender do you identify with?

□ Female
□ Male
□ Other / Prefer not to say

1. How old are you?
   [Open text response]

**Debriefing**

In this study, we are adopting a behavioural science approach to understand the views, barriers and motors faced by physicians, medical practitioners, midwives, nurses, and pharmacists, when considering whether to engage in vaccination advocacy.

This research will inform the development of evidence-based interventions aiming to address key barriers to vaccination uptake and advocacy, thus creating value for healthcare practitioners, and their vulnerable patients.

The survey design and the analysis will follow a procedure previously developed by Prof G Vallee-Tourangeau in the context of flu vaccination (Kassianos et al., 2018; Vallée-Tourangeau et al., 2017).

Thank you for your participation!
